# Anaphylaxis induced by nectarine

**DOI:** 10.1186/2045-7022-5-S1-P12

**Published:** 2015-03-11

**Authors:** Ting Xiao

**Affiliations:** 1 The First Affiliated Hospital, China Medical University, Shenyang, China

## Background

Peach allergy is common. However, anaphylaxis induced by nectarine has rarely been reported.

## Method

Herein, we report a case of anaphylactic shock caused by nectarine. Results: A 70-year-old man presented at the department of emergency with a 30-minute history of generalized flush, hives and itching. He had an over 60 years' history of peach allergy. Because of decreased vision, he had eaten several pieces of nectarine 20 minutes before the onset of the symptoms. Physical examination showed generalized flush, more than 50 confluent wheals, hypotension, tachycardia. He was conscious and afebrile. Anaphylatic shock caused by nectarine was diagnosed. Intramuscular adrenaline 0.3 mg was administered. His symptoms relieved after 30 minutes. Then oral loratadine 10 mg was administered. His family and he was warned to refrain from both peach and nectarine.

## Conclusion

Patients with peach allergy should be watchful on the cross allergic reaction with nectarine.

## Consent

Written informed consent was obtained from the patient for publication of this abstract and any accompanying images. A copy of the written consent is available for review by the Editor of this journal.

